# Metabolic Plasticity of Acute Myeloid Leukemia

**DOI:** 10.3390/cells8080805

**Published:** 2019-07-31

**Authors:** Johanna Kreitz, Christine Schönfeld, Marcel Seibert, Verena Stolp, Islam Alshamleh, Thomas Oellerich, Björn Steffen, Harald Schwalbe, Frank Schnütgen, Nina Kurrle, Hubert Serve

**Affiliations:** 1 Department of Medicine 2, Hematology/Oncology, Goethe University, 60590 Frankfurt am Main, Germany; 2 German Cancer Consortium (DKTK) and DKFZ, 69120 Heidelberg, Germany; 3 Center for Biomolecular Magnetic Resonance, Institute of Organic Chemistry and Chemical Biology, Goethe-University, 60438 Frankfurt am Main, Germany; 4 Frankfurt Cancer Institute (FCI), 60590 Frankfurt am Main, Germany

**Keywords:** acute myeloid leukemia, metabolic plasticity, aerobic glycolysis, TCA cycle, oxidative phosphorylation, amino acids, fatty acids, leukemic stem cells, drug resistance, redox homeostasis

## Abstract

Acute myeloid leukemia (AML) is one of the most common and life-threatening leukemias. A highly diverse and flexible metabolism contributes to the aggressiveness of the disease that is still difficult to treat. By using different sources of nutrients for energy and biomass supply, AML cells gain metabolic plasticity and rapidly outcompete normal hematopoietic cells. This review aims to decipher the diverse metabolic strategies and the underlying oncogenic and environmental changes that sustain continuous growth, mediate redox homeostasis and induce drug resistance in AML. We revisit Warburg’s hypothesis and illustrate the role of glucose as a provider of cellular building blocks rather than as a supplier of the tricarboxylic acid (TCA) cycle for energy production. We discuss how the diversity of fuels for the TCA cycle, including glutamine and fatty acids, contributes to the metabolic plasticity of the disease and highlight the roles of amino acids and lipids in AML metabolism. Furthermore, we point out the potential of the different metabolic effectors to be used as novel therapeutic targets.

## 1. Introduction

Acute myeloid leukemia (AML) is characterized by abnormal proliferation of undifferentiated and non-functional hematopoietic cells (the leukemic blasts) in the bone marrow [1]. The median age of affected individuals is 68 years, and the course of the disease is highly heterogeneous. In many individuals, the disease presents with high blast counts in peripheral blood. In other patients, the appearance of blasts is confined to the bone marrow, with variable proliferative potential. Invariably, AML infiltration causes bone marrow failure, and besides treatment aimed at eradicating the disease, substitution of blood components, as well as prophylaxis and treatment of infections are important therapeutic measures to prevent AML fatality.

On a molecular level, AML develops by the accumulation of multiple genetic and epigenetic alterations in hematopoietic progenitors that are subject to clonal evolution, with the result of considerable inter- and intraindividual heterogeneity of AML clones. Over the last thirty years, the highly complex genomic landscape of AML has been deciphered, starting with the discovery of characteristic cytogenetic aberrations, followed by the discovery of single oncogenic mutations. More lately, systematic sequencing of thousands of AML genomes revealed the full genomic landscape that is characterized by high heterogeneity, clonal evolution, and considerable dynamics of the mutational events over the course of the disease. One of the molecular hallmarks of AML are balanced chromosomal translocations, often resulting in structural aberrations in myeloid transcription factors. Other common mutations aberrantly activate signaling molecules, involving classical oncogenes as for example Ras, or, more AML-specific oncogenic effectors like the receptor tyrosine kinase fms like tyrosine kinase 3 (FLT3), that is mutated in about 30% of the cases. Whole genome analyses have also led to the discovery of mutations in epigenetic modifiers, metabolic enzymes, and in RNA splicing factors, just to name a few. In combination, these mutations provide different aspects of the phenotypic hallmarks of leukemia: limitless proliferation, enhanced stem cell function, and a block of differentiation that arrests the leukemic cells in the proliferative pool of myeloid progenitors ([2,3], reviewed in [4,5]). The dynamic changes in the mutational landscape of AML are the result of clonal evolution, where clones harboring novel mutations are constantly put under selective pressure within the bone marrow microenvironment, with the possible result of loss of a respective clone, clonal persistence, or clonal outgrowth. Clonal evolution does not come to a halt under therapy, which simply provides a change of the selective pressure. Therefore, AML relapse often considerably varies in its genomic setup (and therefore also phenotypically) from the disease at presentation ([6,7], reviewed in [8]). The genomic landscape has been widely used to classify AML. The current World Health Organization (WHO) classification lists several AML entities that are defined by molecular aberrations [9]. Also, the association of molecular aberrations and the response of AML patients to therapy led to the inclusion of these aberrations into sophisticated models to assess patient prognosis, which is important to guide therapeutic decisions. [10,11]. Thus, deciphering the molecular landscape of AML has helped to elucidate the cellular pathogenesis of the disease and to guide treatment decisions according to individual prognosis prediction (reviewed in [12,13]).

Despite these advances, the knowledge of AML driver mutations only rarely led to the discovery of novel drugs, at least until recently. Since about 50 years ago, the combination of cytarabine (Ara-C) and an anthracycline remains the core of AML therapy (reviewed in [14,15]). Allogeneic hematopoietic stem cell (HSC) transplantation is the most important therapy to prevent relapse after initial successful treatment (reviewed in [12,13]). These therapeutic strategies are, however, utterly unsatisfactory: despite significant progress, mainly due to the increased ability to successfully treat opportunistic infectious diseases, to provide supportive care, and to master the complications of allogeneic bone marrow transplantation, the majority of AML patients still die from this disease [1]. Therefore, rational development of novel therapies is desperately needed. In the last two years, several novel therapies entered the stage and started to enrich the armamentarium for the treatment of defined subgroups of AML patients. This progress has been reviewed elsewhere [16,17].

**Figure 1:**
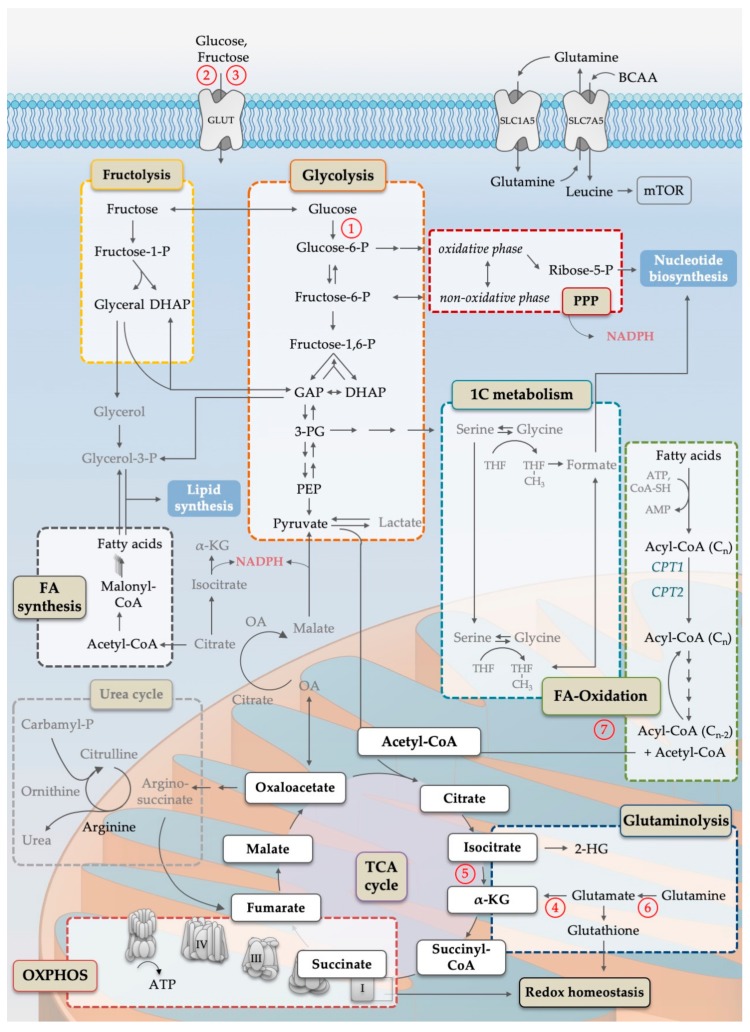
Simplified scheme of the cellular metabolism pathways important for AML pathophysiology. Names in colored boxes indicate major metabolic pathways. Oxidation of glucose in the course of glycolysis (orange box) generates carbon intermediates that can be metabolized in the pentose-phosphate-pathway (PPP, dark red box) and one-carbon metabolism (1C, blue box), both generating substrates for nucleotide biosynthesis. Parts of PPP as well as reactions catalyzed by IDH and malic enzymes are the main producer of cytosolic NADPH. Enzymatic degradation of fructose by fructolysis (yellow box) provides carbon intermediates for the glycolytic pathway as well as substrates for lipid synthesis. Acetyl-CoA, generated by glycolysis, fructolysis or fatty acid β-oxidation (green box), is the starting point for the mitochondrial tricarboxylic acid cycle (TCA, purple circle), which is essential to ensure oxidative phosphorylation (OXPHOS, red box). Acetyl-CoA is used for the condensation reaction of oxaloacetate to citrate. Citrate can be further processed to generate cytosolic acetyl-CoA for fatty acid synthesis (grey box). Furthermore, TCA cycle intermediates, e.g., oxaloacetate, are linked to the urea cycle, an important arginine source. In contrast, anaplerotic reactions, such as glutaminolysis (dark blue box), replenish the TCA cycle. Additionally, glutaminolysis is important for the synthesis of glutathione, a major player in maintaining cellular redox homeostasis. The exchange of glutamine to branched chain amino acids (especially leucine) is known to be a main activator of mammalian target of rapamycin complex 1 (*mTORC1)* signaling, directly linking metabolites to anabolic growth programs resulting in nucleotide, lipid and, in particular, protein synthesis. Single arrows indicate preferred direction of reactions of the main pathways, while multiple arrows indicate multiple reaction steps. Abbreviations: 1C; one carbon; 2-HG, 2-hydroxyglutarate; 3-PG, 3-phosphoglyceric acid; α-KG, alpha ketoglutarate; AMP, adenosine monophosphate; ATP, adenosine triphosphate; BCAA, branched chain amino acids; CoA, coenzyme A; CPT, carnitine palmitoyl transferase; DHAP, dihydroxyacetone phosphate; FA, fatty acid; GAP, glyceraldehyde-3-phosphate; GLUT, glucose/fructose transporter family; mTOR, mammalian target of rapamycin; NADPH, nicotinamide adenine dinucleotide phosphate; OA, oxaloacetate; OXPHOS, oxidative phosphorylation; -P, -phosphate; PEP, phosphoenolpyruvate; PPP, pentose-phosphate pathway; SLC, solute carrier family; TCA, tricarboxylic acid; THF, tetrahydrofolate. Circled numbers refer to metabolic response determinants in AML that are discussed in the Conclusion section.

Leukemogenic genetic and epigenetic lesions fundamentally change the way how leukemic progenitor cells proliferate, differentiate, and interact with the bone marrow microenvironment. Experimental evidence suggests that they also fundamentally change the way how these cells feed and how they process fuels to provide energy and biomass for survival and proliferation (for graphical depiction of major metabolic pathways in AML see Figure 1). It is the hope that interference with these malignancy-inducing and -maintaining metabolic programs may be used for therapeutic intervention. This idea is not novel. In fact, the very first successful trial utilizing chemotherapy for a malignant disease showed that aminopterin, which interferes with the tetrahydrofolate metabolism, can be used as a therapeutic agent in acute lymphoblastic leukemia (ALL), and a derivative of this agent, methotrexate, is until today a core agent in the treatment of this deadly disease [18]. In recent years, cancer metabolism and ways to target it for therapy has been revisited (reviewed in [19,20]). Here, we will focus on the metabolism of AML.

AML is no exception to the general rule that altered utilization of glycolysis is a major hallmark of malignant growth [21,22]. Mitochondrial metabolism including the tricarboxylic acid (TCA) cycle and oxidative phosphorylation (OXPHOS) have been found to drive AML maintenance. Similarly, specific alterations in amino acid metabolism, fatty acid oxidation (FAO) and fatty acid synthesis (FAS) were shown to contribute to AML growth. Interesting recent work links specific leukemogenic driver lesions to metabolic abnormalities. For example, FLT3-internal tandem duplication (ITD) mutations are associated with increased glycolytic activity through the upregulation of the glycolytic gate keeper enzyme hexokinase 2 (HK-2) [23]. Neomorphic mutations in the cytosolic and mitochondrial isoforms of isocitrate dehydrogenase (IDH), IDH1 and IDH2, respectively, result in the formation of 2-hydroxyglutarate (2-HG), an oncometabolite that alters the activity of histone modifying enzymes, and thus the epigenetic landscape of leukemic progenitors [24]. An evolving field of high therapeutic interest is the determination of drug sensitivity by metabolic wiring. For example, recent work shows that an increase in OXPHOS dependency correlates with exquisite dependence of AML cells from the protein B-cell lymphoma 2 (BCL-2) and thus with very good response of the respective patients to a combination of venetoclax, also known as ABT-199, and a hypomethylating agent. The details will be reviewed below. The first papers that systematically screen for response-modifying candidates among metabolically active enzymes are being published [25] and promise to be a valuable resource to formulate hypotheses about rational drug combinations.

Taken together, there is no doubt that in the course of clonal evolution, AML clones in the bone marrow are being trained by their microenvironment to optimally adapt not only to regulatory circuits of cell signaling and gene regulation, but also to optimally adapt to metabolic conditions—displaying a high metabolic plasticity that we only begin to understand.

## 2. Reprogramming of the Glycolytic Metabolism in AML

The exquisite dependence of cancer cells on glucose uptake and utilization is well-known and has been extensively studied. As Otto Warburg described in 1924 [26], cancer cells tend to not utilize the mitochondrial metabolism of the TCA cycle followed by OXPHOS to fully burn glucose-derived pyruvate. Rather, cancer cells convert pyruvate to lactate, which results in low yields of ATP. While it was long thought that this was due to a lack of cancer cell ability, it was more recently appreciated that the Warburg effect is an essential anabolic mechanism that allows cancer cells to master cell growth and division, and that it is caused by oncogenes that hijack growth factor signaling pathways [27]. Several diversions from glycolysis, most importantly the pentose-phosphate pathway (PPP), enable cancer cells to provide nucleotides, amino acids, and electron carriers that are necessary for cancer progression. Thus, cancer cells are highly dependent on glycolysis—not so much for energy production, but rather as a platform to produce building blocks (for a recent review on the complex adaptation processes of glycolysis during tumor progression, see [28]). The Warburg effect ensures a constant high activity of glycolysis and maintains the balance between energy supply and the anabolic functions of glycolysis and its deviations.

### 2.1. Increased Reliance on Glucose Consumption in AML

Evidence suggests that AML cells display high glucose consumption and heavily rely on it. Imaging studies utilizing ^18^F-Fluoro-deoxy-Glucose (^18^FDG) as a marker in 124 AML patients were recently reported by Cunningham et al. (2016). Here, AML bone marrow uniformly displayed high glucose uptake [29]. Herst et al. (2011) reported that high levels of aerobic glycolysis at diagnosis were predictive for improved therapy response and survival of a small series of AML patients [22]. In another study, Chen et al. (2014) compared serum samples from 400 AML patients with those from 446 healthy controls, and showed that sera obtained from AML patients have a distinct glucose metabolic signature exhibiting significant alterations in six metabolites in this pathway [30]. Among those, lactate, 2-oxoglutarate, pyruvate, 2-HG and glycerol-3-phosphate were found to be negatively associated with survival. No significant differences were observed among distinct WHO AML subtypes, suggesting that this metabolic signature may be a consistent feature of AML, independent of cytogenetic risk groups [30]. The authors also analyzed four AML cell lines (U937, OCI-AML3, THP-1 and KG-1), and showed that the expression of various glycolytic and TCA genes was indeed upregulated in comparison to the low-glycolytic cell line HL-60. Incubation with glycolytic inhibitors resulted in decreased proliferation. In addition, knockdown of hexokinase-1 (HK-1) in U937 and OCI-AML3 cells, as well as treatment of the above mentioned AML cell lines and primary AML blasts with the glycolysis inhibitor 2-deoxy-D-glucose (2-DG) increased the sensitivity to the chemotherapeutic agent Ara-C [30].

### 2.2. Cytosolic Carbohydrate Metabolism

Cellular glucose uptake is mediated by transmembrane glucose transporters (GLUTs). High expression levels of GLUT family members was detected in many different cancers, e.g., GLUT1 in Burkitt’s lymphoma, or GLUT2 in hepatocellular carcinoma [31,32,33]. Several papers addressed the expression of GLUTs in AML cells, possible mechanisms of how the expression might be regulated, and analyzed the association of GLUT expression with patient response to therapy and prognosis. In one patient cohort, increased GLUT1 mRNA expression levels were found to be associated with poor responsiveness to chemotherapy [34,35]. Sun et al. (2018) demonstrated that the long noncoding RNA (lncRNA) Antisense RNA at the *INK4* Locus (ANRIL) was upregulated on mRNA level in AML patients of different stages in comparison to normal controls and patients in complete remission (CR). Knockdown of ANRIL led to increased senescence in HL-60 and MOLM-13 cells in vitro, and to increased survival rates upon transplantation of the latter in NOD/SCID mice in vivo. On a molecular level, ANRIL positively regulated GLUT1 protein expression and drove glucose metabolism by a mechanism involving adiponectin receptor 1 (AdipoR1), the cellular energy sensor adenosine monophosphate -kinase α (AMPKα) and sirtuin-1 (SIRT1) [36].

HK-2 catalyzes the first step of glycolysis and has been well described to be activated by phosphoinositide-3-kinase (PI3K)/protein kinase B (PKB/AKT)-dependent mechanisms. The murine lymphoid cell line Ba/F3 showed, upon overexpression of FLT3-ITD (Ba/F3/ITD), high dependence on glycolysis and was sensitive to its pharmacologic inhibition. In addition, the cytotoxicity of the FLT3-inhibitor sorafenib was significantly increased in this cell line model in combination with glycolytic inhibitors, arguing that cells may adapt to FLT3-ITD-driven glycolysis and may be specifically vulnerable because of this [23]. A recent finding on the lncRNA urothelial carcinoma-associated 1 (UCA1) also hints to HK-2 as a possible critical molecule that couples AML oncogenic function with glycolytic adaptation. UCA1 was reported to play an oncogenic role in CCAAT/enhancer-binding protein-α dominant negative isoform (C/EBPα-p30)-positive AMLs [37] and to be involved in chemoresistance of AML cells towards daunorubicin-based therapies [38]. The authors of this study also demonstrated that UCA1 functioned as a competing endogenous RNA (ceRNA) of miR-125a, which in turn positively regulates the expression of the miR-125a-target HK-2 [39]. Another study by Xia et al. (2015) demonstrated that HK-2 is a substrate of chaperone-mediated autophagy (CMA) in AML cells. In CMA, selected proteins carrying a CMA-targeting motif are delivered to the lysosome by their binding to the chaperone Hsc70 and consequently interact with the lysosome-associated membrane protein type 2A (LAMP-2A) [40]. Besides showing that HK-2 is subject to CMA, Xia et al. (2015) also showed that simultaneous inhibition of FLT3 and autophagy leads to activation of CMA and subsequently to cancer cell death under normal nutrient conditions [41].

**Figure 2:**
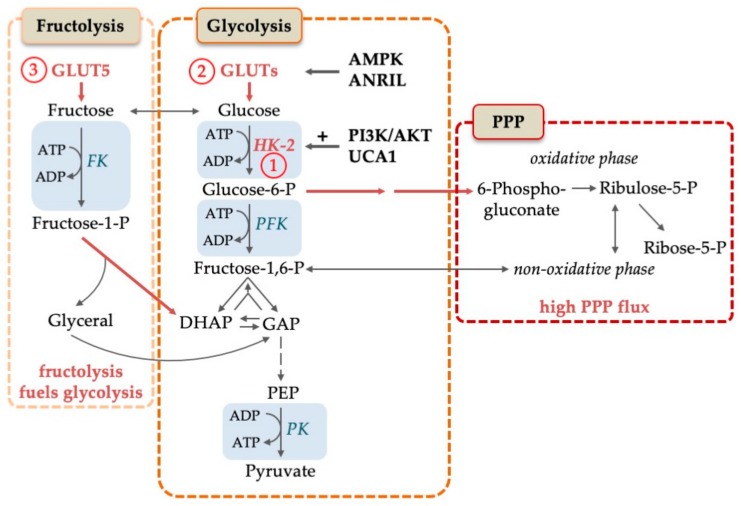
Molecular mechanisms to increase glucose turnover in AML. Glucose is converted to pyruvate in the course of glycolysis. Pentose-phosphate pathway (PPP) and fructolysis are alternative strategies to increase glucose consumption in AML. Fructose is transported into the cell by GLUT5 and consequently phosphorylated by fructose kinase (FK) to fructose-1-P which is funneled into glycolysis. PPP flux is increased in AML, which is needed for the generation of NADPH and building blocks. Rate-limiting steps are represented by blue boxes. Specific alterations in AML, like high expression levels, are shown in red letters and with red arrows. Abbreviations: ADP, adenosine diphosphate; AMPK, adenosine monophosphate activated kinase; ANRIL, Antisense RNA In The INK4 Locus; ATP, adenosine triphosphate; DHAP, dihydroxyacetone phosphate; FK, fructose kinase; GAP, glyceraldehyde-3-phosphate; GLUTs, glucose transporters; HK-2, hexokinase-2; -P, -phosphate; PEP, phosphoenolpyruvate; PI3K/AKT, phosphoinositide 3 kinase/AKT pathway; PKF, phosphofructokinase; PK, pyruvate kinase; PPP, pentose-phosphate pathway; UCA1, urothelial cancer associated 1. Circled numbers refer to metabolic response determinants in AML that are discussed in the Conclusion section.

One advantage of highly active glycolysis for proliferating cells is that glycolysis intermediates can be deviated away from this pathway in order to generate building blocks for biosynthesis. The product of the glucose conversion by HK-2, glucose-6-phosphate (G6P), is entry point into several of such pathways, namely, the PPP (Figure 2), glycogenesis and the hexosamine biosynthetic pathway. Especially the PPP has been studied in AML. Poulain et al. (2017) showed that the PPP functions as an important pro-survival pathway in AML and depends on the activity of the central cellular signaling complex mammalian target of rapamycin complex 1 (mTORC1) [42]. They showed that AML cell lines and primary AML blasts undergo apoptosis upon 6-aminonicotinamide treatment—a potent inhibitor of G6P dehydrogenase (G6PD)—whereas normal hematopoietic progenitor cells were unaffected. Moreover, they claimed that inhibition of mTOR, which was shown to be highly activated in AML [43], leads to a metabolic reprogramming. Indeed, upon simultaneous inhibition of mTOR and G6P, they noted a significant increase in the oxidative metabolism of the TCA cycle, together with resistance to glycolysis inhibitors [42]. These results are in agreement with the already mentioned study by Chen et al. (2014), who also observed a reduction in the PPP intermediate D-ribose phosphate in AML serum samples, which suggests increased activity of the PPP, and of purine synthesis [30]. The importance of the PPP in AML is further supported by the observation that PPP genes are upregulated in 61% of AML patients [44]. Taken together, these results imply that the PPP, and especially G6PD, may define potentially targetable metabolic requirements for AML.

Similar to normal bone marrow, AML hematopoiesis is hierarchically organized, with only a small fraction of AML cells, LSCs, responsible for maintenance of the disease and for relapse after successful treatment. Expression analyses in bulk AML cultures of rate-limiting molecules of metabolic pathways are therefore of limited value for our understanding of important mechanisms of AML, unless they are corroborated by more definitive functional data. Saito et al. (2015) analyzed the effect of AMPK deletion in an MLL-AF9-based in vivo AML model [45]. They found that deletion of AMPK led to a loss of LSCs and to a prolonged latency of AML development upon transplantation. In vitro, AMPK deletion caused loss of GLUTs from the plasma membrane of leukemic progenitor cells, less efficient glucose uptake, reactive oxygen species (ROS) induction and cell death, especially under low glucose conditions.

An interesting and possibly therapeutically targetable variation of the scheme has been reported by Chen et al. (2016) [46]. They show that AML cells can compensate low glucose levels by upregulating *SLC2A5*, the gene for the fructose transporter GLUT5. As depicted in Figure 2, fructose can readily be channeled into glycolysis by fructose kinase. AML cases that display high GLUT5 levels showed a worse prognosis than patients with low GLUT5 levels. Pharmacological inhibition of fructose uptake enhanced Ara-C cytotoxicity in vitro.

A very recent report nicely illustrates, how important sufficient glucose concentrations are in AML. Ye et al. (2018) showed that leukemia cells induce complex manipulations of the host metabolic homeostasis. They showed that AML cells exhibit a tremendous increase (up to 20-fold) in glucose consumption compared to normal hematopoietic cells. The authors further demonstrated that leukemia cells manipulate several host tissues to ensure high glucose concentrations in the bone marrow. The observed effects involve the increase of peripheral insulin resistance and the inhibition of insulin secretion. Interestingly, the molecular mechanisms also involve the establishment of microbial dysbiosis. Therapeutic intervention to relieve the insulin resistance phenotype improved survival and slowed leukemia progression in respective mouse models [47].

### 2.3. Switch between Aerobic Glycolysis and OXPHOS: The Diverging Role in Hematopoietic Stem Cells and Leukemic Stem Cells

In mammals, four pyruvate kinase (PK) isoforms have been described: liver-type PK (PKL), red blood cell PK (PKR) and PK muscle isozyme M1 and M2 (PKM1 and PKM2, respectively) [48]. Cancer cells almost invariably express PKM2 as the predominant PK isoform, and in a landmark paper it was shown that this isoform strongly favors the deviation of pyruvate from the TCA cycle and from OXPHOS [49]. The mechanism is not quite clear yet. However, PKM2 has been shown to display many functions, and its role as a switch between aerobic glycolysis and OXPHOS may well be unrelated to its role as a rate-limiting glycolytic enzyme (for a recent review see [50]). Another potent inhibitor of aerobic glycolysis is the tetrameric enzyme lactate dehydrogenase (LDH) that catalyzes the conversion of pyruvate to lactate. High serum LDH levels at diagnosis are a strong negative prognostic marker for AML patients [51,52].

An important open question in AML research is the importance of the switch between aerobic glycolysis and OXPHOS for LSC function and possibly for the response of AML to therapy. In two remarkable papers, it was shown that the function of normal HSCs critically depends on this switch. For the TCA cycle followed by OXPHOS, pyruvate is channeled into the mitochondrion, where it is dehydrogenated to acetyl-CoA, a prime fuel for the TCA cycle. Pyruvate dehydrogenase (PDH), the enzyme that catalyzes this important step at the onset of the TCA cycle, is phosphorylated and inactivated by several isoforms of PDH kinase (PDK). Using single cell metabolome analyses, Takubo et al. (2013) directly demonstrated that deletion of *Pdk2* and *Pdk4* results in the loss of HSC self-renewal capacity [53]. In the same issue of Cell Stem Cell, Yu et al. (2013) presented the other side of the coin. They showed that conditional deletion of the gene for the mitochondrial phosphatase PTPMT1 resulted in rapid failure of hematopoiesis. PTPMT1 is essential for shuttling pyruvate into the mitochondrion to be utilized as a fuel for the TCA cycle. *Ptpmt1^-/-^* mice harbor a massively increased population of HSCs that lack the ability to commit to hematopoiesis [54]. Thus, in normal HSC, the switch from aerobic glycolysis to OXPHOS is necessary and sufficient to recruit quiescent HSCs into hematopoiesis, and rapidly abolishes the self-renewal capacity of this population. Traditionally, it was thought that metabolic switches in HSCs serve as a response to meet altered requirements of cell fate decisions made by transcriptional, epigenetic and signaling events. Here, it looks as if the metabolic switches themselves determine the fate of HSCs. It remains to be elucidated, how metabolic programs impact cell fate decisions.

The dependence of AML and CML blast crisis LSCs on aerobic glycolysis has been studied by Wang et al. (2014) in MLL-AF9 and BCR-ABL mouse models, respectively [55]. They showed that simultaneous depletion of PKM2 and LDHA, a condition that favors mitochondrial metabolism of pyruvate, strongly inhibited leukemia initiation and maintenance. In this model, the effects of the double knockout on normal hematopoiesis were relatively mild—at least in the absence of HSC stress.

In summary, current data indicate that AML heavily relies on high levels of glycolysis. Thus, a therapeutic intervention to cause a switch from aerobic glycolysis towards mitochondrial respiration might interfere with the growth of AML blasts. Nevertheless, the activity of TCA cycle and OXPHOS, possibly from other fuel sources than pyruvate, seems to be an essential requirement of AML, and will be discussed below.

## 3. Mitochondrial Metabolism: TCA Cycle, OXPHOS and One-Carbon Metabolism in AML Biology and Treatment Resistance

### 3.1. TCA Cycle and OXPHOS in AML

**Figure 3:**
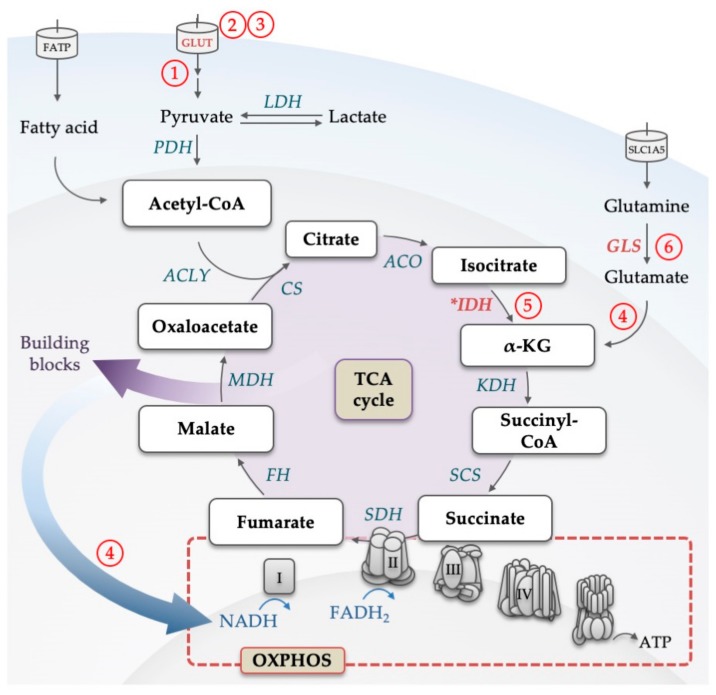
Illustration of the backbone of the tricarboxylic acid (TCA) cycle. Glucose-derived pyruvate and fatty acids are converted to acetyl-CoA, which feeds the TCA cycle. For each glucose molecule metabolized into the cycle, six NADH, two FADH_2_ and two ATP (or two GTP) are being produced. Through the conversion to α-KG, also glutamine can replenish the TCA cycle. Products of the cycle are then utilized in the electron transport chain to produce ATP by the oxidative phosphorylation process. Electrons from NADH and FADH_2_ flow through complexes I, II, III, and IV, which results in a proton gradient across the inner mitochondrial membrane. This in turn provides the energy required to drive ATP synthesis via ATP synthase. Intermediates of the TCA cycle are used as building blocks in other metabolic pathways. Enzymes that are dysregulated in AML are marked in red, mutated are marked with an asterisk. Abbreviations: α-KG, alpha ketoglutarate; ACLY, adenosine triphosphate citrate lyase; ACO, aconitase; ATP, adenosine triphosphate; CoA, coenzyme A; CS, citrate synthase; FADH_2_, reduced flavin adenine dinucleotide; FATP, fatty acid transporter; FH, fumarate hydratase; GLUT, glucose transporter; GLS, glutaminase; IDH, isocitrate dehydrogenase; KDH, α-KG dehydrogenase; LDH, lactate dehydrogenase; MDH, malate dehydrogenase; NADH, nicotinamide adenine dinucleotide; OXPHOS, oxidative phosphorylation; PDH, pyruvate dehydrogenase; SCS, succinyl-CoA synthase; SDH, succinate dehydrogenase; SLC, solute carrier family; TCA, tricarboxylic acid. Circled numbers refer to metabolic response determinants in AML that are discussed in the Conclusion section.

Mitochondria are the central site in the cell, where metabolic pathways that feed from carbohydrates, amino acids and fatty acids (FAs) converge into the TCA cycle and further into an electron transport chain (ETC) that provides energy through OXPHOS (Figure 3).

Preclinical and clinical studies with the compound CPI-613 evaluated the role of the TCA cycle as a potential therapeutic target. CPI-613 is a lipoate derivative that inhibits the enzymes PDH (catalyzing the conversion of pyruvate to acetyl-CoA) and α-KG dehydrogenase (KDH) (converting α-KG to succinyl-CoA). CPI-613 inhibits the oxygen consumption rate in AML cells, and showed evidence of response in a phase 1 trial in patients with diverse hematological malignancies [56]. In a phase 1 study for relapsed and refractory AML patients, where CPI-613 was given in combination with high-dose Ara-C, the regimen showed unexpectedly high remission rates. In accompanying in vitro studies, CPI-613 enhanced the sensitivity of AML cells to Ara-C [57].

To study the role of AML metabolism in chemotherapy resistance, Farge et al. (2017) treated AML patient-derived xenograft-carrying mice with Ara-C and analyzed the resistant cells [58]. They showed that an essential hallmark of Ara-C resistance in AML is the cellular enhancement of mitochondrial metabolism. They showed that AML cells ex vivo have higher mitochondrial density accompanied by high OXPHOS rates. Targeting OXPHOS sensitized the cells to Ara-C and reversed resistance. Consistently, in a recent transcriptome analysis of AML, it was found that enrichment of mitochondrial function genes was negatively correlated with the sensitivity to Ara-C and was associated with a poor prognosis [59]. In another study, enhanced OXPHOS (driven by MYC and MCL1) led to the accumulation of hypoxia-inducible factor 1α (HIF1α) and the development of drug resistance in breast cancer stem cells [60]. HIF1α accumulation was triggered by increased ROS levels, which were in turn due to activated OXPHOS. Inhibition of HIF1α reduced the tumorigenic capacity of those cells and reversed resistance. Although some work clearly hinted towards OXPHOS involvement in driving chemoresistance, further work is needed to investigate the molecular mechanisms. Finally, and most recently, mitochondrial metabolism has been identified as an important response determinant for the promising drug venetoclax, a protein-protein-interaction inhibitor for BCL-2 [25,61]. Venetoclax is registered for the treatment of several lymphoid malignancies. In AML, its clinical efficacy has been demonstrated [62], especially in combination with hypomethylating agents (HMA) and low-dose Ara-C [63,64]. In combination, complete remission rates in previously untreated elderly patients approached up to 67% [64], which may fundamentally change the way, how AML will be treated in the future. In 2018, venetoclax in combination with HMAs was approved for AML patients by the FDA.

In an interesting paper, the Jordan lab analyzed the metabolic state of AML LSCs in comparison to non-LSCs and found that they selectively depended on OXPHOS energy supply. Furthermore, they showed that venetoclax treatment inhibited OXPHOS in these cells, and that venetoclax toxicity was related to these metabolic effects [61]. Similar results were obtained in a recent metabolic loss-of-function screen in AML cell lines. While the most important finding here was that interference with heme synthesis conferred venetoclax sensitivity, the paper also shows that loss of several TCA cycle components, such as the succinate dehydrogenase complex subunits SDHA and SDHC, and proteins enabling the utilization of glutamine as a fuel for the TCA cycle, sensitize AML cells towards venetoclax treatment [25].

In a recent phase 1b clinical study, the combination of venetoclax with the HMA decitabine or azacitidine showed promising synergistic results [63,64]. Here also, the Jordan lab showed that the efficacy of venetoclax was related to selective targeting of the patients’ LSCs ability to perform OXPHOS [65]. Further reports showed that one possible mechanism may be the inhibition of amino acid availability as a fuel for the TCA cycle, on which LSCs from newly diagnosed AML patients exclusively depended [66]. Interestingly, LSCs from AML relapse patients that are more prone to resistance against venetoclax treatment seem to have upregulated mechanisms of FAO and may thus develop resistance against the combination by the acquisition of additional metabolic plasticity.

### 3.2. Maintenance of Mitochondrial Mass and Respiratory Function

One of the basic mitochondrial functions is the transfer of electrons from metabolic intermediates to the final acceptor oxygen. To this end, mitochondria maintain an electrochemical gradient across the inner mitochondrial membrane. A disbalance in the capacity of the ETC to metabolic demands results in the production of ROS. AML cells seem especially vulnerable for this process, because they display a higher mitochondrial mass, and a lower spare respiratory capacity per mitochondrion, rendering them more sensitive than normal hematopoietic cells to oxidative stress [67]. In line with this hypothesis, tigecycline was identified in a chemical screen to specifically inhibit the growth of AML cells but not of normal hematopoietic progenitors [68]. This compound is a highly potent inhibitor of mitochondrial translation with the result that respiratory chain components are further depleted. The authors presented convincing evidence that the antileukemic effects of the drug were connected to this characteristic. A phase I trial with the compound as a single agent in relapsed or refractory AML showed that tigecycline can be given safely. However, the pharmacokinetic profile of the drug was unexpectedly disadvantageous, and no signs of in vivo antileukemic activity was noted [69]. Finally, the group examined yet another avenue to therapeutically target the increased mitochondrial load in AML. They showed that 2’3’dideoxycytidine, which is registered as a reverse transcriptase inhibitor for the treatment of human immunodeficiency viruses (HIV) infection, is specifically toxic to AML cells because it effectively inhibits synthesis of mitochondrial DNA [70].

Cole et al. (2015) [71] identified the mitochondrial ATP-dependent Clp protease proteolytic subunit (ClpP), which interacts with respiratory chain proteins, to be significantly upregulated in a significant proportion of AML patients (45% out of 511 screened AMLs). They showed that ClpP depletion is fatal for ClpP-expressing leukemic cell lines, including the AML cell line OCI-AML2. ClpP depletion deprived the cells from mitochondrial energy production. Interestingly, in the same report, ClpP knockout mice were viable without a phenotype in hematopoiesis. Thus, ClpP is dispensable for normal HSC function. In a xenograft experiment (SCID mice), treatment with A2-32-01, a ClpP inhibitor, reduced the growth rate of the transplanted OCI-AML2 cell line.

Yet another way of mitochondrial quality control is mitophagy, a form of selective autophagy that relies on the expression of the autophagy receptor p62. Through this mechanism, cells have been shown to control the number and to dispose of dysfunctional mitochondria. Loss of p62 hinders clearance of mitochondria from AML blasts and prolongs disease latency in AML mouse models [72]. To keep up just the right number and function of mitochondria is achieved by as yet another regulated mechanism in AML: the active transfer of whole mitochondria from bone marrow stromal cells to AML blasts [73,74]. This process was found to be driven by the enzyme NADPH oxidase-2 (NOX2) that is crucial for the ability of the leukemic cells to form tunneling nanotubes, and has been described also in normal hematopoiesis as a response mechanism to oxidative stress [74]. Depletion of NOX2 in AML cells resulted in reduced respiration capacity, suggesting that this transfer process is essential for energy production in AML blasts. Transplantation of a NOX2 knockdown human AML cell line into NSG mice enhanced survival in comparison to control animals transplanted with NOX2-replete AML lines.

To summarize, due to increased mass of mitochondria with lower ETC capacity and constant oxidative stress, AML cells are at the brink of a failing respiratory system. There seems to be a delicate and incompletely understood balance between the requirement for OXPHOS-dependent energy supply, ROS induced stress, ETC reserve and other mitochondrial functions that is important for proliferation, survival, and cell fate decisions in AML that is very different from normal hematopoiesis. Understanding the regulation of this balance may hold important keys to find AML-specific vulnerabilities.

### 3.3. Metabolic Alterations in AML and Their Influence on the Epigenome

A paradigm of how metabolic pathways may contribute to malignant growth by the alteration of regulatory circuits is illustrated by mutations in the enzyme IDH. Two of the three known mammalian isoforms of this enzyme have been found to be recurrently mutated in AML: the mitochondrial enzyme IDH2 that utilizes NAD+ to oxidize isocitrate to α-ketoglutarate (α-KG) in the TCA cycle, and the cytosolic protein IDH1, that catalyzes the same reaction in the cytosol, utilizing NADP+ and being one of the main cellular producers of NADPH [2]. The mutations are always heterozygote, so that the cell always retains the wildtype function of the enzyme. However, the mutant allele is neomorphic, leading to the production of the aberrant metabolite 2-HG [75]. Figueroa et al. (2010) analyzed a cohort of primary AML samples to understand how metabolic alterations may influence the epigenome and contribute to the evolution of leukemia. *IDH1/2* mutations caused epigenetic modifications in this cohort of patients, leading to global DNA hypermethylation as well as to a specific hypermethylation signature [24]. This in turn was shown to have leukemogenic effects by impairing hematopoietic differentiation. The mechanisms pertaining to how 2-HG changes the epigenetic design have been extensively studied and involve the inhibition of TET2, as well as the complex interference with ataxia telangiectasia-mutated (ATM)-dependent DNA repair mechanisms [76]. Small molecule IDH inhibitors were clinically evaluated in the treatment of *IDH1/2-*mutant AML [77,78]. In both AML patient cohorts, the respective inhibitors ivosidenib (IDH1) and enasidenib (IDH2) resulted in roughly 40% response rates, and about 30% complete remission rates, when given to patients suffering from relapsed or refractory AML as monotherapy. This led to FDA approval of these drugs for this AML patient group that displays the respective mutations.

Similar findings were observed in glioblastoma, in which it was shown that mutated IDH leads to a dramatic elevation in 2-HG, which also changed the DNA methylation status [79]. Interestingly, and possibly not related to the effects of the aberrant metabolite 2-HG on DNA methylation, it was demonstrated by RNA interference screens that *IDH*-mutated AML is especially sensitive to BCL-2 inhibition [80]. This was also shown in vivo, where treatment with venetoclax, reduced the engraftment of primary *IDH*-mutated AML cells transplanted to NSG mice compared to vehicle controls, yet had no significant effect on the engraftment of *IDH1/2* wild-type AML. Evidence was presented that the sensitization effect may be due to 2-HG inhibiting cytochrome c oxidase activity in the mitochondrial electron transport chain, lowering the threshold of BCL-2-induced apoptosis.

The 1C metabolism involving tetrahydrofolate, is the target of the first successful chemotherapeutic agents, aminopterin and methotrexate [18]. The key enzymes for this pathway each exist in a cytosolic and a mitochondrial version. Differences in cytosolic versus mitochondrial NAD(P)+/NAD(P)H ratios drive the pathway into different directions, so that the mitochondrial branch of the 1C metabolism converts serine in association with tetrahydrofolate into glycine and free formate, which is transported to the cytosol, reassociates with tetrahydrofolate, and then serves as a precursor for purine, thymidine and methionine metabolism, which places the pathway into an essential role for nucleotide synthesis and epigenetics (for a review, see [81]). One key step within the mitochondrial branch of this pathway is the oxidation of methyl-tetrahydrofolate to formyl-tetrahydrofolate that is catalyzed by the enzyme methyl-tetrahydrofolate-dehydrogenase 2 (MTHFD2). Gene expression analyses of AML cell lines in response to a variety of antileukemic agents (Vitamin D, ATRA, JQ1, EPZ004777) revealed that MTHFD2 was downregulated by all compounds. MTHFD2 knockdown led to differentiation and hence, proliferation impairment and reduced colony formation capacity in AML cell lines, mouse MLL-AF9 cells and primary AML samples in vitro. Further, MTHFD2 suppression sustained an effect in vivo and reduced leukemia progression in NSG mice [82]. Given the importance of this pathway for the provision of methionine, which is an essential requirement for DNA- and histone methylation reactions, it is tempting to speculate that MTHFD2 might be yet another enzyme that provides a link between AML metabolism and regulation of leukemic growth.

## 4. Amino Acid Metabolism in AML

Amino acids play an essential role in cells, as they are both building blocks for protein synthesis and intermediate metabolites for biosynthetic reactions. Many amino acids have been found to be taken up, synthesized or fed into catabolic and anabolic pathways to a different extent in cancer cells compared to healthy cells reviewed in [83,84,85]. In AML, most data refer to alterations in the glutamine, arginine and branched chain amino acid (BCAA) metabolism.

### 4.1. Pleiotropic Functions of Glutamine

**Figure 4:**
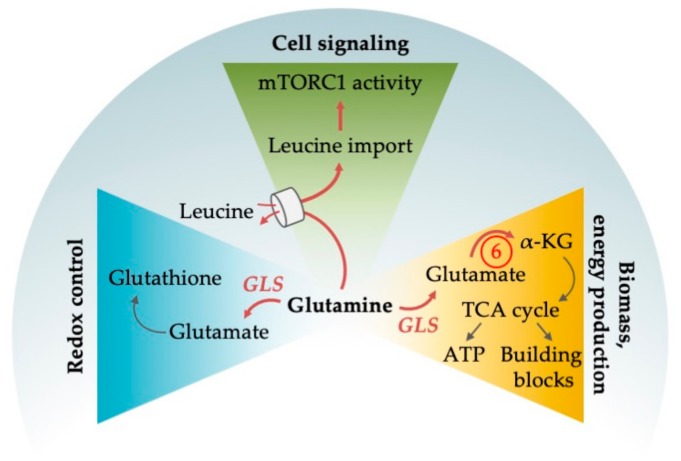
Glutamine plays an essential role in AML for different cellular functions. The amino acid supports redox control by providing the antioxidant glutathione and is involved in cell signaling by indirectly activating the central metabolic regulator mTORC1. Glutamine is further involved in energy production by replenishing the cells’ power hub, the tricarboxylic acid (TCA) cycle. In order to use glutamine both for redox control and energy production, glutamine is broken down into glutamate by glutaminase (GLS) which is found upregulated in AML. Enzymes that are dysregulated in AML are marked in red letters and with red arrows. Abbreviations: ATP, adenosine triphosphate; α-KG, α-ketoglutarate; mTORC1, mammalian target of rapamycin complex 1; NEAA, non-essential amino acids. Circled numbers refer to metabolic response determinants in AML that are explained in the Conclusion section.

The non-essential amino acid glutamine serves as a central node orchestrating a number of cellular functions (for an outline see Figure 4). Glutamine can be produced intracellularly de novo, it can be imported via the glutamine importer SLC1A5, also known as ASCT2, or it can be produced by lysosomal degradation of proteins obtained from autophagy, endocytosis or macropinocytosis. Its central role in metabolism stems from various metabolic pathways that can convert glutamine into α-KG. As a first step, glutamine is converted to glutamate through the enzyme glutaminase (GLS). Glutamate can be directly converted to α-KG via glutamate dehydrogenase (GLUD), or it can be deaminated in a number of reactions and thus utilized as nitrogen source in the production of non-essential amino acids and of purine and pyrimidine nucleotides (for a review on the pleiotropic functions of glutamine in cancer, see [86]). In a process widely utilized by cancer cells and known as anaplerosis, α-KG, as a metabolite of the TCA cycle, can be directly fed into the TCA cycle (Figure 1 and Figure 3). However, α-KG can also be used by the cell as a carbon donor in FAS, in the reduction of NADP+ to NADPH, which is needed as electron donor for the building of macromolecules, and for the production of one of the main ROS scavengers (glutathione) that is especially necessary in cancer cells due to their increased ROS production (Figure 4). Finally, intracellular glutamine levels are central for the regulation of signaling events, most prominently of the activity of mTORC1. Although this central switch between cellular ana- and catabolism is primarily controlled by leucine instead of glutamine, glutamine nevertheless has an important regulatory function here, since the import of one molecule leucine by the amino acid transporter LAT1 requires the export of one molecule of glutamine [87].

#### 4.1.1. Glutamine as an Alternative Fuel for the TCA Cycle

**Figure 5:**
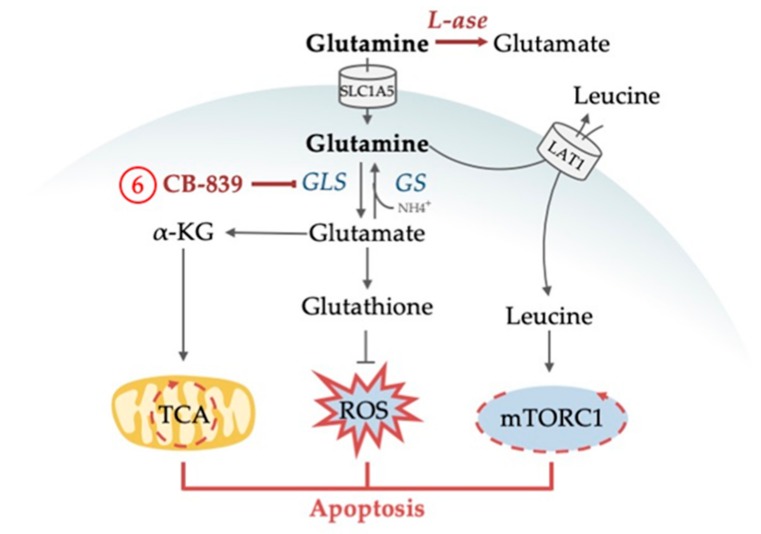
Drug targeting of glutamine metabolism in AML results in the inhibition of central cellular mechanisms followed by apoptosis. CB-839 inhibits glutaminase preventing the utilization of glutamine in TCA cycle replenishment and the production of ROS-eliminating glutathione. L-asparaginase has glutaminase activity and degrades extracellular glutamine. As a result, intracellular glutamine levels are not efficient for leucine uptake and mTORC1 activation. Abbreviations: GLS, glutaminase; GS, glutamine synthetase; α-KG, α-ketoglutarate; L-ase, L-asparaginase; LAT1, large neutral amino acid transporter 1; mTORC1, mammalian target of rapamycin complex 1; NH_4_, ammonia; ROS, reactive oxygen species; TCA, tricarboxylic acid cycle. Circled numbers refer to metabolic response determinants in AML that are discussed in the Conclusion section.

With plasma concentrations of 0.6 to 0.8 mM, glutamine is the most prevalent amino acid in the blood [87,88]. Similar to other cancers, plasma concentrations of glutamine in AML patients are considerably lower with concentrations below 0.3 mM, suggesting high glutamine consumption [88,89,90]. More direct evidence that AML cells are exquisitely dependent on exogenous glutamine came from data, where knockout of the high-affinity glutamine transporter SLC1A5 led to apoptosis of AML cell lines, and prevented tumor development in AML xenotransplantation and primary mouse AML models [91,92]. Several lines of evidence point towards a prominent role of glutamine in anaplerosis in AML. For example, the in vitro toxicity of glutamine deprivation, which is considerable in most AML models, can be rescued by the addition of α-KG to the cell medium, another alternative fuel for anaplerosis besides glutamine [93]. GLS, was shown to be a rate-limiting factor for TCA cycle activity in AML (Figure 3), and was found highly expressed in AML patients [94]. GLS activity can be selectively and irreversibly blocked by CB-839, which has demonstrated antitumor activity across a range of cancers in preclinical studies [95,96]. In AML cell lines, knockout as well as CB-839-induced inhibition of GLS reduced OXPHOS, arrested proliferation and induced apoptosis without affecting normal human CD34-positive progenitors (Figure 5). Interestingly, inhibition of GLS synergized with venetoclax [93]. The effects of CB-839 were abolished by α-KG addition and expression of hyperactive glutaminase C (one of two isoforms encoded by the *GLS* gene), again demonstrating the link between glutaminolysis and TCA cycling that seems essential for AML survival. The effects of CB-839 were also seen in AML cell lines and patient samples harboring *IDH1/2* mutations [94]. Furthermore, CB-839 sensitized FLT3-ITD AML to the FLT3 tyrosine kinase inhibitor AC220 as shown in in vitro and in vivo studies and revealed the metabolic dependency of FLT3-ITD AML on glutamine metabolism [97,98]. Another glutaminase inhibitor, BPTES, was found to specifically suppress cell growth of primary AMLs expressing mutant *IDH,* but not of those expressing wild type *IDH* [99]. Altogether, the data confirmed glutamine and its breakdown in glutaminolysis to be an essential driver of TCA cycle activity and that interfering with the TCA cycle induces robust anti-leukemic responses.

#### 4.1.2. Glutamine Deprivation Affects Redox Control

Oxidative stress and increased ROS production are well-known characteristics of malignant cell growth (reviewed in [100]). AML is no exception to this. ROS production plays a role in the pathogenesis of the disease [101,102]. However, any cell has to elicit an antioxidant response to circumvent cell death induced by excessive ROS production. Glutamine is an important metabolite for the synthesis of the tripeptide glutathione, the prime ROS scavenger (Figure 4). The utilization of glutamine has shown to be essential for redox control in AML [103]. Gregory et al. (2018) showed that CB-839 inhibited glutathione production, that this provoked accumulation of mitochondrial ROS in multiple AML types, and subsequently apoptotic cell death (Figure 5). The combination of CB-839 with drugs that further induced ROS production and mitochondrial apoptosis, such as arsenic trioxide (ATO) and homoharringtonine, enhanced cell death in AML cell lines, primary cells, and in leukemic transplantation models [103]. Recently, the role of the second amino acid of glutathione, cysteine, in redox control was analyzed in LSCs. Depletion of cysteine resulted in breakdown of OXPHOS activity in these cells, as a consequence of ceased production of glutathione with the consequence of impaired SDHA glutathionylation. This reaction is essential for the function of ETC complex II and hence for OXPHOS [104].

#### 4.1.3. Glutamine Deprivation Affects mTORC1 Activity

Cellular leucine directly controls mTORC1 activity. Since leucine uptake is dependent on glutamine, intracellular glutamine concentrations are involved in mTORC1 regulation. This has been shown in AML cell lines, where mTORC1 function critically depends on glutamine availability [91]. In contrast, glutamine starvation did not have any effect on mTORC1 activity in normal CD34-positive cells. L-asparaginase (L-ase), an anti-leukemic agent that also displays glutaminase activity, considerably suppressed mTORC1 and protein synthesis, inducing apoptotic and autophagic responses in primary AMLs (Figure 5). Knockout of glutamine synthetase (GS, catalyzes the condensation of glutamate and ammonia to form glutamine), which was upregulated upon L-ase treatment, further enhanced apoptosis. Such data lead to the revisiting of L-ase preparations, which display considerable glutaminase activity. The agent has well-known antileukemic activity in ALL, where it is one of the backbone drugs for treatment for pediatric and adult patients. Several clinical trials are under way that evaluate novel L-ase preparations for the treatment of AML [105,106,107] and assess its effects on plasma glutamine levels. It will be a considerable challenge for clinical translational research to understand the complex interplay of glutamine metabolism, AML biology, and therapeutic effects of L-ase preparations.

### 4.2. Exploiting the Dependence of AML on Arginine

The majority of research on amino acid metabolism in AML explored the importance of glutamine, yet, a smaller body of studies also described a strong dependence of AML on arginine.

**Figure 6:**
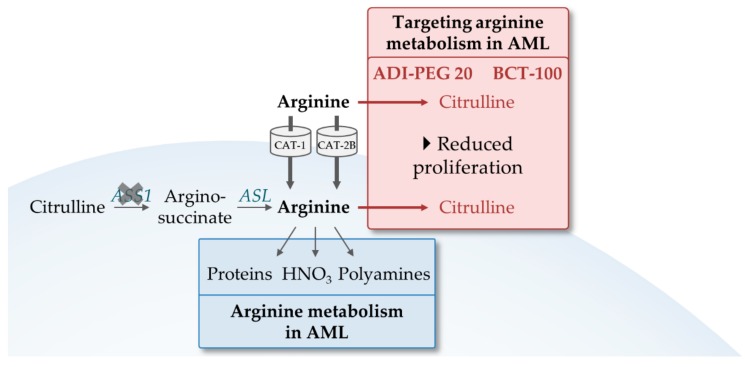
The role of arginine in AML. Arginine is essential in AMLs as the majority lacks argininosuccinate synthetase-1 (ASS1) for de novo synthesis. It is taken up via the transporters CAT-1 and CAT-2B and an important building block for proteins, nitric acid and polyamine synthesis. Upon arginine metabolism inhibition by the arginine deaminases BCT-100 and ADI-PEG 20, cells have a significant reduced proliferation. Red arrows indicate inhibitions by BCT-100 and ADI-PEG 20. Abbreviations: ASL, argininosuccinate lyase; ASS1, argininosuccinate synthetase-1; CAT, cationic amino acid transporters.

Arginine is required by the cells in a number of cellular functions. The amino acid serves as a building block for protein synthesis and is important for the supply of various metabolites including nitric acid and polyamines (Figure 6). Various cancer types have shown an increased dependence on arginine during tumor growth and inhibiting arginine metabolism has been suggested as a potential therapeutic cancer target [108].

Arginine is a conditionally non-essential amino acid, meaning that cells can synthesize the amino acid but under certain circumstances rely on the import of external arginine which is mediated by cationic amino acid transporters (CAT). In order to synthesize arginine, citrulline is metabolized by argininosuccinate synthetase-1 (ASS1) and argininosuccinate lyase (ASL). The majority of AMLs lack ASS1 and therefore depend on exogenous arginine sources that are supplied from the diet [109,110]. Consistently, AML cells constitutively express the arginine transporters CAT-1 and CAT-2B and plasma arginine levels of AML patients are significantly decreased compared to those of healthy volunteers (Figure 6) [110]. Restriction of exogenous arginine resulted in reduced cell viability. The arginine deiminases BCT-100 and ADI-PEG 20 were evaluated for their effects on AML cells in vitro and in vivo. BCT-100 is a human recombinant arginase and has shown to rapidly deplete arginine intra- and extracellularly. The agent reduced in vitro the proliferation of primary AML blasts, and significantly inhibited growth of HL-60 xenografts. [110]. This effect was further enhanced in combination with Ara-C [110]. ADI-PEG 20, a mycoplasma-derived arginine deiminase, also reduced AML burden in sensitive primary AMLs and xenograft models. Here also, ADI-PEG 20 enhanced sensitivity to Ara-C, which also showed synergistic effects in cells that were resistant to ADI-PEG 20 as a single agent due to ASS1 overexpression [109].

All in all, the above referenced studies confirmed that arginine dependence is a metabolic characteristic of AML which is enhanced by the lack of ASS1, the enzyme that allows arginine de novo synthesis. Promising results were reported in early clinical trials with ADI-PEG 20 in lung cancer, in ASS1-deficient malignant pleural mesothelioma, and also in AML [111,112,113]. A clinical trial with BCT-100 is underway that also includes AML patients [114].

### 4.3. Branched-Chain Amino Acids

BCAAs include leucine, isoleucine and valine. Their synthesis is catalyzed in a reversible transamination reaction by the BCAA transaminases 1 (BCAT1) and 2 (BCAT2). In many cancers, BCAAs have been shown to be essential nutrients. Overexpression of BCAT1 has been associated with an aggressive tumor phenotype and disease progression in several cancer types, including breast and liver cancer (for a review, see [115]).

Recently, altered BCAA metabolism and high levels of BCAT1 have nicely been shown to be involved in AML biology. The Trumpp lab [116] showed in an elegant paper utilizing quantitative expression proteomics on sorted populations of primary AML bone marrow samples that BCAT1 was found significantly overexpressed in AML LSCs but not in the non-LSC bulk population. This lab further showed that BCAT1 overexpression resulted in enhanced α-KG amination and thus lowered intracellular levels of α-KG. This induced changes in epigenetically active α-KG-dependent dioxygenases including the EGLN1 and TET family of enzymes, resulting in similar epigenetic changes in BCAT overexpressing LSCs as in *IDH*-mutant LSCs. Indeed, BCAT1 overexpression was associated with a bad prognosis in patients without, but to a much lesser extent with *IDH* mutations. In another paper, Hattori et. al (2017) showed that BCAT1 is upregulated in and important for disease progression from chronic myeloid leukemia (CML) chronic phase to blast crisis, and that it is also important in de novo AML. Metabolomic analyses showed that BCAAs as the products of BCAT1 are indeed the culprit for the effects on malignant growth. A chemical inhibitor of BCAT1, gabapentin, effectively suppressed clonal growth of AML cell lines and primary AML cells [117]. Since the effects of BCAT1 seem LSC-specific and given that gabapentin is readily available for clinical use, it will be interesting to see whether clinical strategies are being developed to exploit BCAT1 as a therapeutic target in myeloid leukemias.

## 5. The Emerging Role of Lipid Metabolism in AML

As mentioned in the previous sections, to meet the exquisite demands on cell growth and proliferation, AML highly depends on the production of biomass and energy. In mammals, lipids are mainly used as a source to produce ATP, NAD(P)H and building blocks for specialized lipids that are essential signaling molecules. Provision of cells with FAs is achieved by transporter-mediated FA uptake, hydrolysis of triglycerides or by de novo FAS. FA consumption is achieved by mitochondrial β-oxidation, also known as FAO, that results in the production of acetyl-CoA, flavin adenine dinucleotide (FADH_2_) and NADH, which feed into the other core mitochondrial metabolic pathways: TCA cycle and OXPHOS. Alternatively, acetyl-CoA from FAO, after conversion in the TCA cycle to citrate, can be transferred to the cytosol and utilized for the production of NADPH (for a review see [118]). In the last few years, it has been shown that FA uptake and consumption mediate important aspects of AML biology with influence on LSC fate decisions, adaptation to a specialized microenvironment, and response/resistance to drugs.

**Figure 7:**
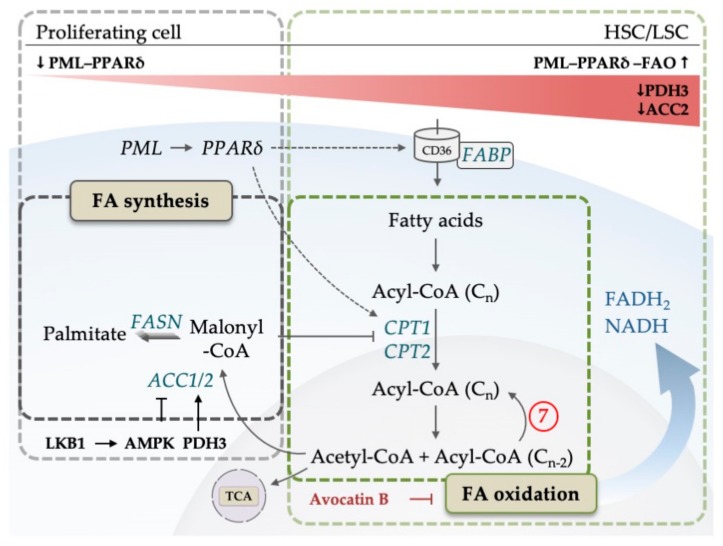
Simplified model of fatty acid (FA) metabolism. Fatty acid synthesis (FAS) starts after acetyl-CoA is converted to malonyl-CoA by ACC. Malonyl-CoA is then further processed by FAS. On the right hand side, fatty acid oxidation (FAO) of acyl-CoA begins after fatty acids are transported into the cell by CD36 and converted to acyl-CoA. The translocation of acyl-CoA into the mitochondria is facilitated by CPT1/2. Avocatin B accumulates in mitochondria to inhibit FAO. While proliferating cells show elevated FAS for the generation of membranes, HSCs/LSCs upregulate FAO to maintain quiescence controlled by PML-PPARδ-FAO axis. PDH3, which is downregulated in AML, promotes FAO by decreasing ACC2 activity. LKB1-AMPK pathway inhibits ACC proteins and promotes FAO. Dotted lines indicate target genes of PPARδ. Abbreviations: ACC, acetyl-CoA carboxylase; AMPK, adenosine monophosphate (AMP)-activated kinase; CoA, Coenzyme A; CD, cluster of differentiation; CPT, carnitine palmitoyltransferase; FA, fatty acid; FABP, fatty acid binding protein; FADH_2_, flavin adenine dinucleotide; FAO, fatty acid oxidation; FASN, fatty acid synthase; HSC, hematopoietic stem cell; LSC, leukemic stem cell; LKB1, liver kinase B1; NADH, nicotinamide adenine dinucleotide; PDH3, Prolyl hydroxylase 3; PML, promyelocytic leukemia; PPARδ, Peroxisome proliferator-activated receptor; TCA, tricarboxylic acid. Circled numbers refer to metabolic response determinants in AML that are discussed in the Conclusion section.

The first step in cellular FA utilization is the uptake of long chain FAs, facilitated by CD36, fatty-acid-binding proteins (FABP) and a number of transport proteins [119]. Once inside the cell, FAs are activated in a two-step reaction catalyzed by acyl-CoA-synthetase to form acyl-CoA. Acyl-CoA is broken down to acetyl-CoA in the process of FAO inside mitochondria. FA oxidation is composed of repetitive cycles of catabolic reactions that result in the shortening of FAs to generate NADH, -FADH_2_- and one acetyl-CoA per cycle (Figure 7). A rate-limiting step of FAO is catalyzed by carnitine palmitoyl transferase 1 (CPT1), which conjugates FAs to carnitine, a prerequisite for mitochondrial translocation of FAs.

The starting molecule for de novo FAS is cytosolic acetyl-CoA, which is carboxylated to malonyl-CoA in a rate-limiting step catalyzed by acetyl-CoA-carboxylase 1 and 2 (ACC1, ACC2) [120]. Subsequently, in each cycle of seven different reactions, catalyzed by the multifunctional enzyme fatty acid synthase (FASN), two carbon units of malonyl-CoA are linked together until the saturated 16-carbon fatty acid palmitate is synthesized (Figure 7). Palmitate is further processed by elongases and desaturases to produce many other (unsaturated) FAs. FAS is linked to the cellular energy balance by AMPK, which can inhibit ACC proteins. Interestingly, ACC is also subject to hydroxylation by a member of the above-mentioned α-KG-dependent dioxygenases from the EGLN1 family, PHD3, which activates ACC2 [121]. Malonyl-CoA, the product of the reaction that is catalyzed by ACC, inhibits CPT1 and thus mitochondrial FAO.

Fatty acid metabolism has been shown to be heavily involved in the regulation of HSC cell fate. Analysis of the promyelocytic leukemia protein (PML) function revealed a signaling system that in addition to PML involves PPARδ and FAO. Here, Ito et al. (2012) showed that PML controls the extent of asymmetric (HSC-preserving) versus symmetric (HSC-exhausting) HSC division via the activity of the transcriptional nutrient sensor and regulator of the FAO transcriptional program, PPARδ (for a review of PPARs in regulating FA metabolism, see [122]), in a fashion that is dependent on FAO [123]. Liver kinase B1 (LKB1), a regulator of AMPK, is essential for normal HSC function, an effect that is at least partially dependent on the FAO-promoting effect of LKB1 that regulates the PGC-1 coactivators of the PPAR family of nuclear receptors [124].

First evidence that FA metabolism may play an important role in AML came from the Andreef lab (2010), which showed that pharmacological inhibition of CPT1 (inhibiting the mitochondrial translocation of FAs) enhanced AML sensitivity towards apoptosis-inducing agents that interfere with the mitochondrial apoptosis machinery. Their data indicated that this may be due to a specialized function of FAO that is not related to ATP production. Rather, FAO serves to regulate the BAK-dependent mitochondrial permeability transition, a core function in cytochrome c-dependent apoptosis regulation [125]. Many data point in the direction that AML cells, and especially LSCs depend on high FAO rates and low FAS activity. The above-mentioned α-KG-dependent dioxygenase PHD3, that regulates ACC2 (the rate-limiting FAS enzyme that switches FAS on and FAO off), is down-regulated in AML [121]. AML cells are provided with enough fuel for FAO, so that they are not required to switch on FAS. For example, in a mouse CML blast crisis model, CD36-positive LSCs reside in a specialized niche in gonadal adipose tissue, where they are protected through FAO from cytotoxic therapy and LSC exhaustion [126]. The existence of a CD36-positive LSC fraction with unique metabolic features was confirmed in patients. In a direct assessment of chemotherapy resistance mechanisms in primary AML cells, mice harboring patient-derived xenografts from AML patients were treated with Ara-C and the resistant cells were analyzed for their metabolic profile. Besides a strong predominance of OXPHOS and high ROS levels, the resistant AML cells also showed significant membrane overexpression of CD36, the FA transporter, and increased levels of FAO [58].

A similar observation with regard to FAO was made in venetoclax resistance. As discussed earlier in this review (see Section 3.1), venetoclax sensitivity is enhanced in AML cells that display high OXPHOS—fueled by amino acids. One striking difference between venetoclax-responsive de novo disease and venetoclax-resistant relapsed AML cases was enhanced FAO in the relapse cases. Thereby, FAO could contribute to a general mechanism in therapy resistance. However, it has to be noted that neither the viability nor the colony-forming potential of LSCs was affected by lipid depletion in general [66]. In co-culture experiments, AML cells feed from bone marrow adipocytes with the result that in the AML cells AMPK is activated, and subsequently a transcriptional program is induced that favors proliferation and FAO [127]. In contrast, abrogating intracellular free fatty acid transport into the mitochondria of AML cells resulted in a prolonged survival in AML mouse models [128].

Therefore, a picture emerges where feeding of AML cells with FA is an important requirement for leukemic growth and therapy resistance against cytotoxic agents and apoptosis induction. Cellular uptake of FAs via CD36 and shuffling of FAs into mitochondria with the help of CPT1 are important aspects of this metabolic wiring. FAO feeds into the mitochondrial pathways of TCA cycle and OXPHOS, which seems to be an important mechanism for the maintenance of self-renewal in normal HSCs as well as in LSCs—as long as mitochondrial metabolism is not fed by the main product of glycolysis, pyruvate. However, which aspects downstream of FAO are decisive for leukemic growth are not well understood. One explanation could be that FAO is an important source for NADPH, through the mitochondrial production of acetyl-CoA and citrate that in the cytosol can be utilized by a series of enzymatic reactions to produce NADPH (reviewed in [118]). The relevance of FAO for NADPH homeostasis in cancer cells was described by Jeon et al. (2012). Under metabolic stress, NADPH generation by the PPP is impaired, and increased AMPK levels promote the production of FAO-derived NADPH by decreasing NADPH-consuming FAS [129]. FAO-derived NADPH may well be the decisive cellular electron donor for AML cells under therapy to fight oxidative stress and to serve in anabolic processes that are necessary for cell division.

Evidence that this may indeed be the case comes from interesting work regarding Avocatin B. Screening a natural product library on selective leukemic cell toxicity, Lee et al. (2015) identified Avocatin B as a potent anti-leukemic agent in vitro. Avocatin B is an odd-numbered carbon lipid derived from avocado fruit. It was shown to inhibit FAO resulting in a 50% reduction of NADPH levels followed by ROS-mediated apoptosis. The activity of Avocatin B was abolished in leukemic cells lacking CPT1 [130]. When AML cells were co-cultured with bone marrow adipocytes and treated with Avocatin B [131], cells adapted by the induction of fatty acid binding protein 4 (FABP4) production and increased glucose and FA uptake, presumably as a consequence of AMPK and activating transcription factor 4 (ATF4) activation. At the same time, ROS levels increased, and the cells became exquisitely sensitive to Ara-C treatment.

## 6. Conclusions

Through clonal evolution, leukemic hematopoiesis in AML constantly adapts to environmental conditions. Over the last years it became clear that this not only involves adaptive changes in signaling and transcriptional control, but also the way in which the leukemic cells feed and how they metabolize. The environmental conditions provide the selection pressure for this adaptation, and we have seen significant changes in the dependence of AML cells on carbohydrates, amino acids and lipids, and significant changes in the way how these metabolites are processed within the preformed circuits of metabolism. Seemingly, the large body of literature is confusing, and the experimental approaches are hampered by presumed heterogeneity of the metabolism of LSCs versus bulk AML blasts, of AML cases and models driven by heterogeneous oncogenes, and by the necessary reduction of complexity in AML models.

However, the work overwhelmingly suggests that during the evolvement of the disease, AML cells considerably gain metabolic plasticity, which enables them to successfully infiltrate the various bone marrow niches, outgrow and annihilate normal hematopoiesis. Enhanced consumption of metabolites that are available at comfortable concentrations like glucose [29,30,47], glutamine [88,89,90,91,92,93] and FAs [121,125,126,127] is one gain that helps AML cells to divide more rapidly than their normal counterparts, and to evade the commonly encountered apoptotic stimuli. AML cells rewire the metabolic pathways so that they can optimally use the food without being at loss in energy or building blocks. Although there is still enough room for speculation, the Warburg effect is one example of how AML cells like any cancer cell make sure that the high glucose consumption is not wasted to swamp the cell in energy and leaving it at loss of the products of other pathways like nucleotides and NADPH from the PPP [26]. However, since the meaning of any metabolic rewiring will be highly dependent on the cellular and environmental context, it is evident that the above-mentioned disease heterogeneity ([2,3], reviewed in [4]), and the specificities of the disease models limit the extent to which we can pinpoint with high confidence the functional role of metabolic pathways or even of single metabolic enzymes. Therefore, this line of experiments may need much more time until it can help us to rationally decide on targets for future AML therapy. Nevertheless, it will be very interesting to further see how AML cells, or specific subpopulations, differ in their metabolic wiring from normal cells, and how they rebalance, for example, in response to the enhanced ROS production that results from oncogenic stress.

On the other hand, the enhanced metabolic plasticity comes at a risk for AML cells. Clonal evolution is extremely efficient to optimize cellular metabolism for specific selective conditions. A sudden change of the selective pressure, for example, by therapeutic interventions, calls a new game and may turn a selective advantage into a lethal threat for the cell. This principle of synthetic lethality is not restricted to metabolic wiring. However, recent and also not so recent evidence suggests that it applies here, and that it can be clinically successful. It is arguably so that aminopterin and methotrexate worked in acute leukemias because of this principle, clinical evidence that Sydney Farber published in 1948 [18]. Recently, the principle has—as reviewed—very nicely been demonstrated by showing that high OXPHOS activity fueled by amino acid anaplerosis may support leukemic growth, but it also renders AML sensitive to combined treatment of venetoclax with an HMA [66]. As summarized in Table 1, systematic analyses of metabolic determinants of drug response in AML have just begun, and it will be very interesting to see further results, which may allow us to find novel prediction markers for specific drugs to stratify patients accordingly. Since, in contrast to most driver lesions of AML, metabolic enzymes are relatively easy targets for pharmacological intervention, the hope is that these results will also point the way towards treating patients with modifiers of metabolism that force the AML metabolism into a sensitive state towards antileukemic drugs from cytotoxic agents to targeted small molecules.

Since clonal evolution does not stop with the start of therapy, but rather is a common mechanism for the advent of secondary resistance, it will be important to eradicate the disease rapidly, and not to give it time to adapt. This has also been appreciated by hematologists for decades. However without a real clue as to which patients and why the initial attempt to eradicate the disease failed, despite the use of highly effective drugs like Ara-C and daunorubicin, that can reduce the leukemic cell burden in most patients in the order of several log steps. While it has been widely thought that most of the relapses stem from LSCs that somehow survive the initial onslaught of cytotoxic drugs, the reason why they might do so is not well understood. Some papers strongly suggest that it may be metabolic states that deviate in LSCs and especially in resistant LSCs from the bulk of sensitive AML blasts [61,66,116], highlighting that future analyses should focus on the LSC-positive population to identify LSC-specific vulnerabilities. Again, a recent discovery along this line relates to venetoclax, where resistant AML blasts seem to have acquired the ability for fatty acid anaplerosis, which may circumvent venetoclax/HMA vulnerability that characterizes AML cells that depend on amino acid anaplerosis [66]. Systematic search and characterization of metabolic differences between AML blasts at diagnosis and at relapse, and the development and application of single cell metabolic analyses are eagerly awaited to help design treatment strategies aimed at hitting LSCs while saving HSCs.

It is exciting times for research aimed at AML metabolism. Patients desperately need novel ideas for how to treat this disease.

## Figures and Tables

**Table 1:** Reviewed possible metabolic response determinants in AML. Numbers (No) correspond to the circled numbers indicated in the respective figures.

No	Figures	Description	References
1	1, 2, 3	Hexokinase is involved in resistance against FLT3-ITD inhibitors	[23]
		Hexokinase inhibition increases sensitivity against cytarabine	[30]
		Hexokinase is involved in resistance against daunorubicin	[38]
2	1, 2, 3	Glucose transporter (Glut1-Glut4) expression decrease chemotherapy sensitivity	[34,35]
3	1, 2, 3	Inhibition of the fructose transporter Glut5 enhances cytarabine cytotoxicity	[46]
4	1, 3	Amino acid consumption in oxidative phosphorylation may be associated with venetoclax sensitivity	[65,66]
5	1, 3	Mutated IDH1/2 are clinically validated targets for AML therapy	[77,78]
6	1, 3, 4, 5	Inhibition of glutaminase (GLS) synergizes with venetoclax and with AC220	[93,97,98]
7	1, 3, 7	Fatty acid-oxidation may be related to venetoclax resistance	[66]
